# Separation Anxiety, Attachment and Inter-Personal Representations: Disentangling the Role of Oxytocin in the Perinatal Period

**DOI:** 10.1371/journal.pone.0107745

**Published:** 2014-09-17

**Authors:** Valsamma Eapen, Mark Dadds, Bryanne Barnett, Jane Kohlhoff, Feroza Khan, Naomi Radom, Derrick M. Silove

**Affiliations:** 1 School of Psychiatry, University of New South Wales, Sydney, New South Wales, Australia; 2 Academic Unit of Child Psychiatry, South West Sydney Local Health District, Liverpool, New South Wales, Australia; 3 School of Psychology, University of New South Wales, Sydney, New South Wales, Australia; 4 Karitane, Villawood, New South Wales, Australia; 5 Centre for Population Mental Health Research, Psychiatry Research and Teaching Unit, South West Sydney Local Health District, Liverpool, New South Wales, Australia; Catholic University of Sacred Heart of Rome, Italy

## Abstract

In this paper, we aimed to assess cross-sectionally and longitudinally associations between disturbances in maternal early attachment experiences, symptoms of separation anxiety and depression and oxytocin plasma levels. We examined a mediational model that tested the hypothesis that anxious attachment style arising from the mothers’ early bonding experiences with her own parents was associated with high levels of separation anxiety which, via its impact on depression, was associated with reduced levels of oxytocin in the postnatal period. Data is reported on a structured sample of 127 women recruited during pregnancy from a general hospital antenatal clinic and an initial follow up cohort of 57 women who were re-assessed at 3-months post-partum. We found an association between lower oxytocin level in the post partum period and symptoms of separation anxiety and depression during pregnancy, as well as maternal negative interpersonal representations, upbringing attributes and anxious attachment style. Further meditational analysis revealed that the unique association between anxious attachment and depression is mediated by separation anxiety and that depressed mood mediated the relationship between separation anxiety and oxytocin. In conjunction with evidence from the literature suggesting that lower oxytocin level is associated with bonding difficulties, our findings have significant implications for understanding the biological processes underpinning adverse attachment experiences, negative affect state, and mother-to-infant bonding difficulties.

## Introduction

There is converging evidence that oxytocin plays an important role in early bonding and parent-infant interactions, a process that exerts a profound impact on the later development, competencies and mental health of the child [1], [2]. Studies have shown links among maternal depression, suboptimal bonding behaviours and lower levels of oxytocin in the blood. We extend this area of inquiry by considering the possible mediating role of separation anxiety, a common reaction pattern in pregnant mothers, which is known to be associated with insecure attachment styles.

Oxytocin is a neuropeptide hormone produced by neurons in the supraoptic nucleus and the paraventricular nucleus which project to limbic sites including the amygdala, ventral striatum, hypothalamus, nucleus accumbens and the mid-brain [3]. It is thought that the hormone is instrumental in the formation of parent-infant bonding and in establishing and maintaining social affiliative behaviours [4] through a bio-behavioural feedback loop. Oxytocin is central to the process of motivating approach and engagement with others; it increases attention to, and the accurate perception of, salient social information; and in addition improves social recognition. These processes are all essential in the formation of secure maternal-infant bonds [5].

There is emerging evidence that disruptions in oxytocin function are associated with impairments in social functioning and affiliative behaviours, including maternal bonding to infants [6], [7]. In humans, mothers whose oxytocin levels exhibit a pronounced peak around parturition report the greatest attachment to their unborn babies [8] and following birth, demonstrate more attentive maternal behaviours, namely eye gaze, ‘motherese’ vocalisations, positive affect and affectionate touch [8]. Taken together, the accruing evidence provides growing support for the role of oxytocin in the process of maternal-infant bonding.

The quality of the mother’s own early experiences from her parents may influence her ability to provide sensitive care to her infant. In animal studies, for example, adult female rats who received good maternal care show quicker and more responsive caregiving behaviours and an associated increase in oestrogen-induced oxytocin receptor binding in specific brain areas [9]. The role of oxytocin in mediating the impact of the mother’s own early experiences is supported by experiments showing that rat pups given oxytocin show greater maternal caring behaviours as adults while caring for their offspring than conspecifics administered an oxytocin antagonist as infants [10]. Further, animal studies suggest that central oxytocin receptors are pruned on the basis of the quality of maternal care provided [9], [11], which may reflect a process of early epigenetic programming. Hence, if generalised to humans, the existing evidence suggests a direct relationship between the quality of parental bonds in the mother’s early life and her future central oxytocin response and caring capacity in relation to her bonding with her infant.

There is still uncertainty about the central oxytocin responses in human mothers. The majority of research with human participants has been conducted by taking peripheral measures of endogenous oxytocin or using exogenous administration of oxytocin (e.g., nasal sprays) [12]. The relationship between central and peripheral oxytocin is currently under debate [13] as are the methods of oxytocin administration and collection [12]. Nevertheless, the relationship between peripheral oxytocin and maternal mood during the perinatal period has received increasing attention [12]. Plasma oxytocin levels in the third trimester of pregnancy predict risk of postpartum depression. It is noteworthy that both postpartum depression and decreased oxytocin are associated with impairments in maternal bonding [13]. Whether the relationship between maternal depression and oxytocin is direct or indirect remains to be established however, given that there are a range of other psychological disturbances in pregnancy that are associated with disturbed maternal-infant bonding. In particular, anxiety is common in pregnancy and there is some evidence that the affective state including anxiety impacts adversely on the capacity of the mother to interact with her infant [2]. To date, however, evidence is somewhat inconsistent concerning the relationship between maternal anxiety and oxytocin. Studies have varied in showing either an inverse relationship between anxiety and oxytocin [14], no relationship between the two [15] and an indirect association modulated by attachment [16] and/or moderated by gender [17]. One of the complexities is that there may be a bidirectional relationship between peripheral oxytocin and anxiety: oxytocin exerts anxiolytic effects but is also released in response to stress [18].

It is possible that particular subtypes of anxiety may have specific associations with both maternal attachment difficulties and oxytocin levels. We have found that separation anxiety is a common form of anxiety amongst pregnant women [19] and that it often presents with comorbid depression [20]. Separation anxiety is characterized by fears for the safety and well-being of close attachment figures and/or dread that harm will befall these persons. As would be expected, there is a relationship between high levels of separation anxiety and an anxious or insecure style of attachment [21]. It seems likely therefore that women experiencing high levels of separation anxiety in pregnancy are at risk of encountering difficulties bonding with their infants. Separation anxiety shows a pattern of comorbidity with depression in pregnancy [22]; thus, one possibility is that separation anxiety mediates the effect of an anxious attachment style in leading to depression and decrements in oxytocin levels in the perinatal period. Further, it has been observed [23] that the change in oxytocin level across the pregnancy to postpartum period can predict maternal-foetal bonding, with lower levels associated with lower maternal-foetal bonding scores and deficient maternal behaviours.

This study aimed to evaluate the association between early maternal attachment experiences, symptoms of separation anxiety and depression and oxytocin plasma levels during pregnancy and the early postpartum period. We examined a mediational model that tested the hypothesis that anxious attachment style arising from the mothers early bonding experiences with her own parents was associated with high levels of separation anxiety which, via its impact on depression, was associated with reduced levels of oxytocin in the postnatal period.

## Methods

This research was fully approved by the South West Sydney Local Health District Human Research Ethics Committee (SWS-LHD HREC). All participants gave written informed consent prior to participation.

### Participants

The study consisted of 127 participants purposively sampled from a larger pool of pregnant women (total *n* = 668) attending a general hospital antenatal clinic between June 2011 and May 2013. All women attending the antenatal clinic who were aged 18 years or over (at the projected time of delivery), English speaking, less than 38 weeks of gestation, and pregnant with a singleton infant were invited to take part in a longitudinal study investigating maternal separation anxiety using the Adult Separation Anxiety Questionnaire (ASA-27)[24]. In order to allow comparisons with other studies examining anxiety in pregnancy and the unique contribution of separation anxiety to maternal bonding and related experiences, participants were allocated to the “separation anxiety group” if they scored over the threshold (i.e., total ASA-27 score >22) for clinically significant symptoms or to the “without separation anxiety group” if they scored below threshold. The sample was progressively targeted to include sufficient, balanced number of participants allocated to with and without separation anxiety groups. In the final five months of the recruitment period (Jan 2013–May 2013), participants were included in the sample for follow up if they scored over the threshold (i.e., total ASA-27 score >22) for clinically significant symptoms given target numbers had been reached for the “without separation anxiety group”.

Data are reported on the initial cohort of 127 women assessed during pregnancy and the 57 women who at the time of sending the first batch of samples for oxytocin analysis had completed study measures at 3-months postpartum. It is also noted that 117 women from prenatal assessment are continuing participation in the study and their postpartum oxytocin levels will be assessed at a future follow-up period, allowing a test of the replication of our findings in due course. Overall, ten participants recruited to the cohort dropped out or were lost to follow up. Women who took part in both stages of the study tended to be less advanced in pregnancy at the time of recruitment (*M* ± *SD* = 19.0±5.4 weeks) than the larger pool participating only in stage 1 (*M* ± *SD* = 22.2±6.6 weeks) (*t* (125) = 3.03, *p*<.05). There were, however, no other significant differences between the groups in terms of demographic characteristics or scores on key study variables.

The average age of study of the cohort (*n = *127) at the time of recruitment was 28.88 years (*SD* = 6.12, range = 17–47) and most (75%) were in the second trimester of pregnancy (*M ± SD* gestation in weeks = 20.44±6.15, range = 8.4–38). Eighty six per cent were married or in a de facto marital relationship and 38.6% were primigravid. The majority (84%) had completed secondary school education and one third had a university degree. More than a quarter were working in full-time employment and around 41% were engaged in full time care-giving. The sample was ethnically diverse, with around half (52%) coming from a Caucasian background, with the remainder coming mostly from Asian (23%), Indian (10%) and Arabic (8%) backgrounds.

### Procedure

At prenatal stage of the study (during pregnancy), participants completed three self-report questionnaires; the Edinburgh Postnatal Depression Scale (EPDS) [25], the State and Trait Anxiety Inventory (STAI) [26] and the Adult Separation Anxiety Questionnaire (ASA-27) [24], and were asked to provide blood samples for oxytocin determination during their next antenatal blood collection at either 28 weeks (*n* = 97) or 32 weeks gestation (*n* = 22), 8 participants with pregnancy in advance of 32 weeks had blood collected before 39 weeks gestation. At postpartum stage (at 3-months postpartum), participants gave a blood sample for plasma oxytocin analysis and completed the EDS and the ASA-27, plus the Attachment Style Questionnaire (ASQ) [27], the Mother-to-Infant Bonding Scale (MIBS) [28] and the Measure of Parental Style Questionnaire (MOPS) [29]. The participants were informed about the blood collection on arrival and they spent about 30 minutes completing the questionnaire following which the blood was collected using a venepuncture on the cubital fossa of the arm.

### Measures (supplementary information)

#### Edinburgh Postnatal Depression Scale (EPDS; [25])

The EPDS is one of the most widely used self-report measures to screen for depression during the perinatal period. It comprises 10 items (e.g., “I have felt so unhappy that I have been crying”), each rated on a four-point scale as experienced over the past seven days. The scale has been validated for use in antenatal population, and a cutoff score of 13 or more suggested as indicative of probable depression [27] with a sensitivity of 68% and a specificity of 96%, for minor and major depression combined, in a community sample of women ([25]; [30]).

#### State and Trait Anxiety Inventory (STAI; [26])

The STAI [26] consists of 40 items divided into two subscales, state anxiety, which assesses current anxiety symptoms (e.g., “I am tense”) and trait anxiety, which assesses general symptoms (e.g., “I am a steady person”). Items are rated on a four-point scale and added to yield total subscale scores. Cut-off scores of above 40 have been found to yield the greatest sensitivity and specificity in antenatal samples [31]. The STAI has been found to have high internal consistency with alpha coefficient ranging from 0.86 to 0.95 [26].

#### The Adult Separation Anxiety Checklist Scale (ASA-27 [24])

The ASA-27 is a self-report questionnaire designed to assess separation anxiety symptoms in adulthood. The ASA-27 contains 27 items, each answered on a four-point scale. Scores can be summed to yield a total score, with higher scores indicating greater separation anxiety symptom severity. The measure has sound internal consistency (Cronbach’s alpha of 0.95).

#### Attachment Style Questionnaire (ASQ; [27])

The ASQ is a 40-item self-report questionnaire designed to measure an adult’s attachment to others and Feeney et al [29] reported α coefficients for the scale that ranged from.76 to.84. Items such as “I worry about people getting too close” are rated on a six-point scale. Items load onto two higher-order factors, an ‘*avoidance* of intimacy’ and an ‘anxious attachment (*anxiety* about abandonment)’ representation.

#### Mother-to Infant Bonding Scale (MIBS;[28])

The MIBS is an 8-item self-report measure designed to assess a mother’s feelings towards her baby during the early postpartum period. Each item consists of an adjective (e.g., “resentful” or “protective”) and is rated on a four-point scale. MIBS has been shown to satisfactorily detect difficulties in mother-child bonding with a sensitivity of 0.9 and a specificity of 0.8 for a threshold score ≥2 [34]. The Cronbach’s alpha for the MIBS in the current study was 0.91.

#### The Measure of Parental Style (MOPS; [29])

The MOPS is a self-report measure designed to assess representations of parenting style during the first 16 years of life. Respondents are asked to rate each parent on 15 items (e.g., “overprotective of me” and “uncaring of me”) using a four-point scale, yielding total scores for each parent on subscales labelled ‘indifference’, ‘abuse’ and ‘over-control’. The Cronbach’s alphas for the indifference and abuse subscales in the current study (mother and father scales averaged) were.88, and.92 respectively.

#### Plasma oxytocin extraction and radioimmunoassay (RIA)

The radioimmunoassay (RIA) method on extracted samples was used in this study as this is one of the best available methods for plasma oxytocin estimation [12]. The RIA method has been developed using independently derived antisera and independently conducted validations. In most cases, validation included evaluation of cross-reactivity with known peptides and proteins (e.g., vasopressin and neurophysin), demonstration of the need to extract samples to eliminate interference and to concentrate the low levels of oxytocin present in plasma, evaluation of the extraction method employed (e.g., recovery of radiolabel and/or recovery after spiking) and in several instances, confirmation of the RIA results by comparison to bioassay [32] including in pregnancy and parturition [33] and or with chromatographic separation [34], [35]. In spite of this methodological diversity, these independently developed and validated RIAs have produced consistent results thereby making it an efficient and accurate method of peripheral oxytocin determination. Blood samples were collected into vacutainer tubes and samples were centrifuged at 3000 RPM for 10 min. Supernatants (plasma) were pipetted and stored at −20°C until extraction was performed. At the time of extraction 20 mg heat-activated (700°C) LiChroprep Si 60 (Merck) in 1 ml distilled water was added to each sample, mixed for 30 minutes and centrifuged. The pellet was washed with distilled water and 0.01 N acidic acid, and then mixed in 60% acetone to elide the neuropeptide, and evaporated extracts were kept at −20°C. The 0.05 ml of assay buffer was added and oxytocin assessed using a highly sensitive and specific radioimmunoassay. To each aliquot 0.05 ml antibody and 0.01 ml l-labeled tracer were added and after an incubation period of 3 days unbound radioactivity was precipitated by activated charcoal. All evaporated plasma extracts to be compared were treated identically. The detection limit is in the 0.5 pg/sample range and antiserum cross-reactivity of less than 0.7%. The samples were analysed at the Max Plank Institute of Psychiatry, Munich, Germany [36].

### Statistical analysis

To examine demographics and scores on key study variables, descriptive statistics and Pearson’s correlations were conducted using SPSS, version 19 for Windows. To examine the unique contributions of attachment representations and affective status during pregnancy on postpartum peripheral oxytocin level, direct and mediated relationships between attachment style (ASQ –anxiety subscale), adult separation anxiety (ASA-27 score during pregnancy), depressive symptoms during pregnancy (EDS score during pregnancy) and oxytocin levels at 3-months postpartum were tested using AMOS v.21 with maximum likelihood estimation to model missing data. For all analyses, alpha was set at *p* = .05.

### Ethics statement

The study was approved by the Institutional Human Research Ethics Committee. All participants gave written, informed consent prior to participation. Deidentified data are made available by contacting the primary author.

## Results

### Results during pregnancy

Table 1 shows the mean (*SD*) scores of the main study variables, at both stages of the study. During pregnancy, the proportion of women displaying clinically significant depression, state anxiety, trait anxiety and ASA symptoms were 13%, 25%, 31% and 44%, respectively. Scores on each of these variables were positively correlated (*r* = .59, *p*<.001). At 3 months postpartum (stage 2), the proportions of women displaying clinically significant depressive symptoms and ASA symptoms were 14% and 26%, respectively. The correlation between these variables at stage 2 was also significant (*r = *.69, *p*<.001). As also shown in Table 1, peripheral oxytocin level during pregnancy showed no association with any of the key study attachment or symptom indicators. The influence of the duration of gestation on oxytocin level was tested between samples collected at 28 weeks and 32+ weeks which showed no significant difference (*t*(125) = -.554, *p* = .24).

**Table 1:** Bivariate correlations between key study variables during pregnancy and oxytocin levels at 3 months postpartum.

Variable	Mean (SD)	Correlation with OXT duringpregnancy (n = 127)	Correlation with OXT at 3-monthspostpartum (n = 57)
Prenatal			
Adult Separation Anxiety (ASA-27 total)	18.98(14.51)	.11	−.32*
Depression symptoms (EDS total)	5.84(4.97)	.09	−.34**
State Anxiety (STAI-State)	30.25(10.33)	.07	−.45**
Trait Anxiety (STAI-Trait)	33.33(10.38)	.07	−.47**
Postnatal			
Adult Separation Anxiety (ASA-27 total)	16.75(12.74)	.16	−.34*
Depression symptoms (EPDS total)	5.37(5.37)	.20	−.36**
Attachment anxiety (ASQ-Anx)	2.94(.63)	.03	−.35**
Attachment avoidance (ASQ-Av)	2.87(.69)	.06	−.42**
Paternal Indifference (MOPS-F-I)	1.27(2.45)	−.08	−.27
Paternal Abuse (MOPS-F-A)	1.54(3.62)	−.14	−.28*
Paternal Over-control (MOPS-F-O)	2.69(2.19)	−.08	−.16
Maternal Indifference (MOPS-M-I)	1.82(3.36)	−.02	.05
Maternal Abuse (MOPS-M-A)	1.58(2.96)	−.04	.08
Maternal over-control (MOPS-M-A)	3.75(2.98)	−.08	.02
Mother to Infant Bonding (MIBS)	1.46(2.22)	.02	−.33*

*p<.05 (2 tailed), **p<.001 (2 tailed).

### Results three months post-partum

When assessed at 3 months postpartum, significant associations emerged between oxytocin level and ASA, depression and anxiety subscale score with those having higher scores of ASA, anxiety and depression showing significantly lower levels of oxytocin. There was also significant association between attachment styles and oxytocin levels as measured by the ASQ with a positive association observed on the subscale of confidence and negative association with subscales of discomfort with closeness, need for approval and preoccupation with relationships. Regarding attachment representations, significant negative associations were found between oxytocin level and both avoidant and anxious attachment. Further, evaluation of mother’s own upbringing using the Measurement of Parenting Style (MOPS) found a negative association between oxytocin level, “father indifference” and “father abuse”. Similarly scores on the Mother-to-Infant Bonding scale were negatively correlated with oxytocin level. Thus, lower levels of peripheral oxytocin level measured at 3 months postpartum were significantly associated with symptoms of ASA, depression and state/trait anxiety during pregnancy, and with ASA and depressive symptoms, attachment insecurity (anxiety and avoidance), recollections of paternal abuse during childhood, and poorer mother-infant bonding at 3 months postpartum.

### Mediation analysis

**Figure 1:**
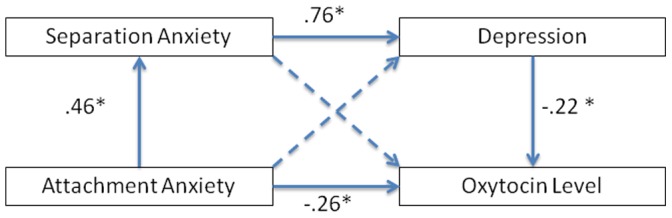
Direct and mediated relationships at postpartum between early anxious attachment, mental health (separation anxiety and depression) and oxytocin levels. Note. Dashed lines represent non-significant pathways; solid lines indicate significant pathways using one-tailed z-tests of regression weights.

As shown in Figure 1, we further examined the unique contributions of early anxious attachment, adult separation anxiety, and depression at 3 months postpartum, using mediation analysis with maximum liklihood estimation in AMOS v.21. This revealed a significant direct effect whereby anxious attachment was found to be associated with lower oxytocin levels. Anxious attachment was also associated with higher levels of adult separation anxiety, which in turn was associated with higher levels of depressive symptomatology, the latter being related to lower oxytocin levels postpartum. Thus, it appears that adult separation anxiety mediated the relationship between anxious attachment and depression, and depression mediated the relationship between separation anxiety and oxytocin.

## Discussion

This study aimed to evaluate the association between early maternal attachment experiences, separation anxiety, depression and oxytocin plasma levels during pregnancy and the early postpartum period. Our key finding was that anxious attachment in pregnancy had both a direct and indirect association with oxytocin postpartum, the latter pathway being mediated via separation anxiety and depression. These findings suggest that the inherent attachment style of the mother and its relationship with symptoms of separation anxiety are central to the impact of depressed mood in relation to the pattern of oxytocin release. Thus it would seem that in the context of a securely-attached female with low separation anxiety, oxytocin release, a key factor shown by past research in regulating the mother’s response to stress, assists in promoting positive affiliation with supportive attachment figures and in shaping optimal bonding with her infant [37]. While it is possible that breastfeeding is associated with an increase in oxytocin levels, previous research has indicated that there are no differences in oxytocin between breast-feeding and non-breast-feeding mothers when samples are not taken during actual breast-feeding [38] since oxytocin production is not pulsatile when mothers are not nursing [39].

The combination of anxious attachment style and separation anxiety may have multiple interacting consequences however, leading to maternal rumination and anxiety as well as distress in relation to interpersonal relationships. It has been shown that such anxiety in turn may generate even greater levels of distress and elevated or protracted stress responses [40], perhaps mediated by the hypothalamo-pituitary-adrenal (HPA) system. In this scenario, elevations in affiliative need may interact with insecure attachment styles, maladaptive interpersonal coping responses, and unsupportive relationships leading to or maintaining depressed mood, and resulting in lower levels of oxytocin.

It is noteworthy that oxytocin release is thought to diminish anxiety by reducing amygdala activation and dampening HPA activity, thus playing an anxiolytic role in the regulation of mood and social stress [2]. Our finding that lower oxytocin level is linked to depression as well as anxiety, including separation anxiety, seems to suggest that decrements in this key hormone may correspond with a negative affective state in general. Given the expectedly high level of comorbidity of different components of anxiety, it is difficult to determine therefore whether separation anxiety per se, or a general vulnerability to emotional disturbance is the key factor associated with lower oxytocin levels. In either event, our findings, coupled with the notion that lower oxytocin level is associated with poor bonding and maternal behaviours, has significant implications for understanding the biological processes underpinning negative affect and bonding difficulties in the perinatal period. In this regard, reduced peripheral oxytocin response has been documented in a range of relevant samples including amongst mothers with lower maternal-foetal attachment scores [23], postpartum depression [41], and cocaine-addiction [12]. Further, oxytocin release has been demonstrated to be inversely related to stress and plasma cortisol levels [42]. At this stage, however, enough is not known about the exact nature and the direction of the relationship between oxytocin and the HPA axis [43].

While maternal caregiving behaviours such as nursing and grooming are similar across individuals, there are also distinct individual attributes based on bonding and attachment which is the cognitive representation of the relationship. There are two global aspects of maternal attachment: the first concerns the formation of a unique and selective bond, while the second relates to preoccupations with infant safety [44]. The latter has been linked to postnatal depression and premature birth [45]. Our finding of an association between lower oxytocin level and anxious attachment representation suggests that these cognitive representations might influence the information processing of affective responses in the context of interpreting infant cues, and these signals in turn may influence oxytocin production. In the infant, these attachment-eliciting signals act as cues to central oxytocin release and receptivity to oxytocin production. In this regard, studies in female voles have shown that hypothalamic oxytocin gene expression and receptor binding typically increases in the postpartum period [46], with deficits in maternal behaviours including lower pup retrieval and less licking and self grooming in oxytocin knockout mice [47]. Cross-fostering studies indicate that individual differences in oxytocin receptors in female pups are related to variations in early maternal behaviour that these pups received as infants as well as the amount of maternal postpartum behaviours such as licking, grooming, and arched back nursing they provided to their infants.

In humans, peripheral oxytocin has been linked to empathy, closeness, and trust [48] and mother-infant touch and contact has been shown to stimulate oxytocin release [49]. Thus, oxytocin seems to play a role in bonding by lowering stress, increasing trust, and integrating psychological and physiological state of calmness and approach [37]. This is also in keeping with the findings that oxytocin response is linked to traits such as more positive and rewarding behaviours, emotions and physical sensations when interacting with infants [50].

Specifically this study found that oxytocin was negatively associated with effortful control and positively associated with orienting sensitivity. This is supported by our finding of a positive association between oxytocin level and maternal confidence and a negative association with discomfort with closeness, need for approval and preoccupation with relationships. Thus, it appears that, when interacting with their infants, mothers with secure attachment representations and positive relationship attributes would produce more oxytocin and through the direct projection of the oxytonergic system to the ventral striatum and dopamine release [51] they would perceive the interaction as more rewarding. The lower levels of oxytocin in mothers with negative cognitive representations of attachment relationships may result in negative maternal behaviours and consequent adverse effects on the infant’s oxytocin receptivity and oxytocin production. This may then lead to difficulties in attachment, including affect regulation, during childhood and ongoing problems with relationships and maternal behaviours in adulthood.

Our finding of a link between lower oxytocin level and mother’s upbringing, characterized by father abuse and indifference, would merit further exploration. In this regard, a study of the role of oxytocin in fathering [52] found that, mothers and fathers showed similar baseline levels of oxytocin. Nevertheless, mothers showing high affectionate contact (caress, kiss, pat, stroke) had increased oxytocin following interaction with their infant, whereas the level was not increased in low affectionate contact mothers or during stimulatory contact. Fathers on the other hand showed oxytocin increase following high levels of stimulatory contact (quick strokes, touching the infant with objects) but not affectionate contact. Thus it is plausible that mothers who would not have had the opportunity for paternal stimulatory contact during their childhood may have lower levels of oxytocin. Further, animal studies indicate that infants reared by low-licking and low-grooming mothers show alterations in brain glucocorticoid receptors [53] and they benefit less from environmental enrichment [54] and similar findings have been observed in children of mothers with postnatal depression [55]. The early intervention strategies should therefore take into consideration the benefits of maternal affectionate contact and paternal stimulatory contact. In this regard maternal affectionate contact involving maternal-infant-skin-to skin contact (kangaroo care) in the neonatal period to promote bonding following premature birth [45] has been described.

Our findings suggest that underlying anxious attachment will manifest overtly as symptoms of separation anxiety which in turn impacts on the mood. Further, as evidenced by the mediational analysis, it appears that the unique association between anxious attachment and depression is mediated by separation anxiety and that depressed mood mediated the relationship between separation anxiety and oxytocin. The association between lower oxytocin level and symptoms of depression coupled with the observation that lower oxytocin level is associated with negative interpersonal representations, upbringing attributes and anxious attachment style has significant implications for understanding the biological processes underpinning negative affect state and bonding difficulties in the perinatal period. Our finding of an association between lower oxytocin and subjective ratings of poorer mother to infant bonding represents an additional finding of our study. It is important to develop a theoretical model to explain this link in order to direct research towards establishing possible mechanisms underlying this association.

### Limitations

This study was limited in its use of peripheral rather than central oxytocin concentrations. There is a debate surrounding whether and how peripheral measures of oxytocin relate to central measures of oxytocin [56]. Given that our findings implicate a role for dysregulated OT biology in attachment and interpersonal difficulties, it is possible that the associations noted in our study population reflect dysregulation in the peripheral oxytocin system which may not adequately represent central brain oxytocin systems. However, available evidence suggests that a coordinated release of oxytocin into central and peripheral areas occurs. For example, the stimulation of axonal projections has been shown to lead to a release of oxytocin into the brain and the periphery [57]–[59]. Functional studies have also shown that peripheral and central concentrations of oxytocin are simultaneously raised during forced swimming stress [60]. While future studies would benefit from assessing the relationship between concomitantly collected blood and cerebrospinal fluid samples to more precisely determine the inter-relationship between the two systems, a significant limitation of studying central oxytocin is that it involves invasive procedures that render them impractical for examining the effects of oxytocin on humans in the social context and in response to environmental events.

## Conclusion

Available evidence from the literature in conjunction with our findings suggests that the oxytocin level underpinned by oxytocin receptivity is a relatively stable trait that reflects an individual’s habitual mode of affect, attachment representations and interpersonal relationship style which in turn has a significant impact on pro-social behaviours and parenting style. Priming of the maternal caregiving system through infant-interaction builds upon the neurobiological basis of oxytocin receptivity laid down during mother’s infancy. Epidemiological studies show that early exposure to dysfunctional parenting is the single most significant (known) risk factor for childhood and later-onset mental disorders including depression, anxiety, disruptive behaviour, and substance abuse disorders [61]. Thus addressing maternal separation anxiety and improving maternal sensitivity and bonding within the early period can alter the trajectory of development through programming of the infant oxytocinergic system and therefore facilitate secure attachment, emotional resilience and positive relationships, mediated by neural receptivity to oxytocin release. Given the central role and the apparent importance of oxytocin to both maternal (and paternal) and infant systems, further research is needed in which data on the biological and family/environmental systems can be integrated longitudinally, starting from pregnancy, and where both parents and the offspring are studied.
